# The Relationship between Health Consciousness and Home-Based Exercise in China during the COVID-19 Pandemic

**DOI:** 10.3390/ijerph17165693

**Published:** 2020-08-06

**Authors:** Bo Pu, Lu Zhang, Zhiwei Tang, Yanjun Qiu

**Affiliations:** 1 College of Tourism, Sichuan Agricultural University, Chengdu 611830, China; 2 School of Public Affairs and Administration, University of Electronic Science and Technology of China, Chengdu 611731, China; tangzw@uestc.edu.cn; 3 Department of Strategy and Discipline, Southwest Jiaotong University, Chengdu 611756, China; publicqiu@vip.163.com

**Keywords:** health consciousness, home-based exercise, health life goal, perceived behavioral control, COVID-19

## Abstract

During the COVID-19 pandemic, people have reduced the frequency of going out, and need to engage in health behaviors at home. Home-based exercise has aroused people’s attention. This paper aims to examine the influencing mechanism of health consciousness on home-based exercise during the COVID-19 pandemic. A questionnaire method was used to select 449 Chinese respondents on an online platform; the questionnaire includes a health consciousness scale, health life goal scale, perceived behavioral control scale, and the home-based exercise scale. A *T*-test was used to conduct differential analysis. The hierarchical regression analysis method was used to examine the relationship between health consciousness and home-based exercise, and the Hayes’ SPSS PROCESS macro was used to test mediating effect. The results show that there are significant differences in home-based exercise with respect to gender, age, and marital status. Health consciousness has a significant positive effect on home-based exercise. Perceived behavioral control acts as the mediator between health consciousness and home-based exercise. Health consciousness can influence home-based exercise through health life goals and perceived behavioral control in turn. This paper takes a home-based exercise survey, and expands the theoretical research of home-based exercise. The findings suggest that people should pay attention to promoting the transformation of health consciousness into home-based exercise. It provides enlightenment for people to adopt health behaviors during the COVID-19 pandemic.

## 1. Introduction

The year 2020 has seen the outbreak of COVID-19, a major global health crisis. As of August 3, 2020, the COVID-19 pandemic has spread to 216 countries, with 17,660,523 confirmed cases and 680,894 deaths [1]. The pandemic has caused a strong impact on the global economy and threatened people’s lives seriously. In this crisis, health has been the top priority. Various countries have actively adopted strict quarantine measures to control the spread of the pandemic. However, the mandatory prohibition of going out has seriously affected people’s daily life [2,3]. It may lead to a decrease in physical activity [2], an increase in sedentary time [4], and then cause physical and mental health problems [4,5,6,7]. Lippi et al. consider that acute cessation of physical activity to prevent the COVID-19 pandemic may cause unfavorable cardiovascular consequences [8]; According to Wang et al.’s investigation [9], in the early stages of the COVID-19 pandemic in China, about a third of respondents reported moderate to severe anxiety. Therefore, during the quarantine, it is particularly important to take measures to improve personal health.

Exercise is one of the important means for people to pursue health. Exercise can be done outdoors or indoors. During the pandemic, the home was the main place of activity. We hypothesize that home-based exercise enters people’s lives. Studies have shown that regular physical activity is an important way to stay healthy during the quarantine [3,8]. Home-based exercise is a convenient and safe way to promote people’s health during the pandemic. For one thing, it can keep people at a healthy level. On the other hand, it can effectively keep coronavirus away by avoiding close contact between people [2,6]. How to promote people’s home-based exercise is worth pondering. However, there are few studies on the influencing mechanisms of home-based exercise during the pandemic.

Health consciousness is the degree to which individuals care about their health [10,11]. The more health-conscious people are, the more likely they are to have healthy habits [12], which is the basis for individuals to take health measures. In recent years, scholars have focused on the influence of health consciousness on various health behaviors, mainly in a healthy diet [13,14,15,16,17,18] and health information [19,20,21,22]. Home-based exercise is a health behavior. Based on this, health consciousness is likely to have an important impact on home-based exercise. However, at present, scholars seldom associate health consciousness with home-based exercise. Simultaneously, the pandemic of COVID-19 made people deeply feel the fragility of life and the importance of health. How to turn the consciousness in the brain into action is very important. Therefore, it is necessary to deeply explore their relationship.

This paper mainly explores a theoretical model, namely, the influencing mechanism of people’s health consciousness on home-based exercise during the pandemic of COVID-19. In this regard, this paper studies from the following aspects. This paper expounds the positive influence of health consciousness on home-based exercise, respectively analyzes the mediating effect of health life goal and perceived behavioral control on health consciousness and home-based exercise, and discusses the mediating role of health life goal and perceived behavioral control between health consciousness and home-based exercise.

The rest of the paper is as follows. The second section reviews the literature and develops hypotheses. Next, we introduce the research process and methods. The fourth section expounds the results. In the fifth section, we conduct discussion including the theoretical contributions and practical implications. The sixth section accounts for limitations and suggestions for future studies, and the final section concludes this paper.

## 2. Literature Review and Research Hypothesis

### 2.1. Health Consciousness and Home-Based Exercise

Consciousness is described as “the state of understanding and realizing something” [23], which is closely related to individual thoughts, memories, feelings, etc. It can not only transmit information, but also produce meaningful information, and constantly change according to individual needs, thus enabling us to act autonomously [24]. Health consciousness is a part of individual consciousness. Becker et al. defined health consciousness as “the degree to which a person is inclined to take health actions” [25]. It has the characteristics of sensitivity to physical health, stress and health hazard factors [26]. Health consciousness contains multiple aspects, which Gould divided into four dimensions: health self-consciousness, health involvement, health alertness, and health self-monitoring [27]. Hong believes that health consciousness includes three potential dimensions: health self-consciousness, personal responsibility, and health motivation [28]. Exercise plays an important role in the personal pursuit of health [29]. A large number of studies have shown that exercise has a positive impact on people’s physical and mental health [30,31,32,33]. Regular exercise can improve immunity, delay aging, reduce fat, and improve cardiopulmonary function [33]. In addition, exercise has a positive effect on the reduction of depression in different groups, such as the elderly [31], students [30], and pregnant women [32]. Home-based exercise, as the name implies, is a related exercise program at home. It can overcome the limitations of the environment [34], provide a cheap, safe and controllable experience [35], and is an important setting for increasing exercise [36,37].

According to the theory of self-consciousness, self-consciousness can predict the consistency of attitude and behavior [38], which extends to health consciousness and health behaviors. There is consistency between health consciousness and health behaviors. Health-conscious people tend to have a better understanding of health themselves, pay attention to individual health problems, and then take healthy measures to ensure their personal health [39]. There are many kinds of health behaviors, such as eating a healthy diet, working out and exercising. Among them, regular exercise is regarded as one of the most beneficial health behaviors [39]. During the pandemic of COVID-19, in the face of the virus, people deeply felt the importance of health to individuals, and their health consciousness would be greatly improved, so they would adopt health behaviors to protect their health. Since activities are confined within the home, these health behaviors often occur within the home, as does exercise. Based on this, the paper assumes that:

**Hypothesis** **1.** *During the pandemic of COVID-19, health consciousness had a significant positive effect on home-based exercise*.

### 2.2. Health Life Goal as a Mediator

Life goal is an ideal state that people pursue, maintain, or avoid [40]. Life goals are a complex hierarchy, divided into external life goals (i.e., money, fame, and image) and internal life goals (i.e., growth, relatedness, helpfulness, and health) [41]. Health life goal is people’s expected goal of pursuing physical health and avoiding diseases. It fully reflects people’s strong desire to keep healthy.

Generally speaking, people with higher health consciousness tend to set many health-related goals in their lives, such as how much time they exercise each day and when they go to bed at night. Once health life goal is set, the motivation to engage in health behaviors will also increase [42]. According to the self-determination theory, life goal orientation has many health benefits, and it has positive effects on health behaviors, life satisfaction, and mental health problems [43,44]. Yuri et al. developed a life goal setting technology, which successfully improved the physical fitness of the frail elderly through three months of exercise [45], and this life goal is a health life goal. The health life goal is the key that life goals have an impact on health behaviors. Therefore, during the COVID-19 pandemic, health-conscious people have a higher health life goal, which leads to a more motivated and planned home-based exercise behavior. Based on this, the paper assumes that:

**Hypothesis** **2.** *Health life goal mediates between health consciousness and home-based exercise*.

### 2.3. Perceived Behavioral Control as a Mediator

Perceived behavioral control is a person’s estimation of how difficult it is to perform a certain behavior [46]. Freud thinks that both conscious and unconscious processes are helpful for the control of human behavior [47]. Hommel believed that consciousness is related to the cognitive function of preparing for relevant actions [48]. The more people know something within their power, the more control they have over it. Loureiro and Breazeale found that consumers’ online shopping tendency has a significant positive impact on perceived behavioral control, and convenience consciousness is one of the most important aspects of online shopping tendency [49]. Thus, consciousness can play a certain role in perceived behavioral control. Similarly, the stronger people’s health consciousness is, the stronger they have the belief that they can control their health, and the lower the probability that their health will be affected by irresistible events [50]. Health consciousness plays a positive role in perceived behavioral control.

Perceived behavioral control is used to predict intentions or behaviors in various fields, including education, psychology, and business, etc. In the field of health, perceived behavioral control is strongly associated with positive health outcomes. Bailis et al. found that socio-economic status has an impact on health self-assessment through perceived behavioral control [51]. Searle et al. significantly improved the quality of life of the elderly by controlling interventions [52]; perceived behavioral control is one of the decisive factors for people to adopt oral health behaviors [53]. It has a significant positive effect on consumers’ purchase of organic meat [16]. In addition, investigators found that perceived behavioral control was the strongest determinant of a healthy diet in young people [54]. Perceived behavioral control has a significant positive effect on health behaviors [16,51,52,54], and home-based exercise is one of the important health behaviors. During the COVID-19 pandemic, health-conscious residents had greater perceived behavioral control, which in turn promoted home-based exercise. From this, the paper assumes that:

**Hypothesis** **3.** *Perceived behavioral control mediates between health consciousness and home-based exercise*.

### 2.4. Health Life Goal and Perceived Behavioral Control

In the theory of planned behavior, behaviors refer to goal-oriented behaviors, and perceived behavioral control is often operated as the possibility of successfully implementing target behaviors [55]. According to this theory, perceived behavioral control can help predict the realization degree of goals [46]. When we set the goal, we will generate a sense of movement [56], and then take target behaviors. Therefore, during the COVID-19 pandemic, health-conscious people often set a series of health life goals, including exercising at home, to generate motivation to achieve health behaviors. Based on this, the paper assumes that:

**Hypothesis** **4.** *Health life goal and perceived behavioral control have a chain mediating effect between health consciousness and home-based exercise*.

**Figure 1:**
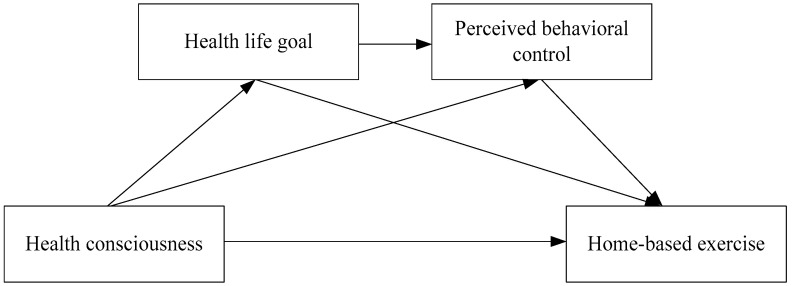
Conceptual model.

According to the above discussion, the conceptual model of this paper is shown in Figure 1.

## 3. Methodology

### 3.1. Scale Development of Home-Based Exercise

#### 3.1.1. Formation of Home-Based Exercise Scale

Currently, there is no international home-based exercise scale for this pandemic. To develop home-based exercise scale during the COVID-19 pandemic, this study took four steps.

Firstly, we referred to relevant studies and scales. Home-based exercise is often discussed by scholars as an intervention to study the effects of home-based exercise on specific populations [57,58]. It varies according to needs, and there is no unified measurement tool to measure home-based exercise. Expanding the scope from home-based exercise to exercise, there were two scales measuring people’s physical activity, the International Physical Activity Questionnaire [59] and the Minnesota Leisure Time Physical Activity Questionnaire [60], which both contain specific exercise items. It was concluded that people could do gymnastic sports, walk or jog, and do housework at home.

Secondly, a survey was conducted. We distributed a survey through “Credamo”, which is a professional platform for online questionnaire design, pushing questionnaires, sample collection, etc. The questionnaire mainly included participants’ demographic information and survey purpose, and those who agreed to participate filled in their contact information in the feedback. We distributed 63 questionnaires, and 50 Chinese residents agreed to take the survey. The response rate was 79%. A total of 22 (44%) participants were male, and 28 (56%) participants were female; 15 (30%) participants were under 25 years old, 18 (36%) participants were between 26–40 years old, 13 (26%) participants were between 41–60 years old, and 4 (8%) participants were over 60 years old; 12 (24%) participants were students, 21 (42%) participants were staffs, 8 (16%) participants were retirees, and 9 (18%) participants engaged in other occupations; 27 (54%) participants came from cities, and 23 (46%) participants came from rural areas. We used voice calls or WeChat text messages to take the survey. The topic of this survey focused on what exercise items they might or have been doing at home during the pandemic; for example, “did you exercise at home during the pandemic?” “What kind of exercise do you usually do?” Based on each respondent’s conversation, We have obtained five aspects of home-based exercise by sorting out, that is, gymnastics (e.g., dance, yoga, aerobics, etc.), walking or jogging, stretching exercises (e.g., leg lift, joint movement, leg press, etc.), housework (e.g., cooking, cleaning, etc.), and other activities (e.g., jumping rope, lifting weights, tai chi, etc.).

Thirdly, an initial scale was formed. According to previous literature and a survey, there are five aspects of exercises that people can do at home during the COVID-19 pandemic, and five items were formed, such as, “during the prevention of COVID-19, I often do gymnastic sports (e.g., dance, yoga, aerobics, etc.) at home”. Further, an expert panel (comprising two professors and a lecturer) evaluated the five items, and we added the item “during the prevention of COVID-19, I do some exercises at home” according to the suggestion of the expert panel, which made the scale more complete.

Fourthly, the initial scale was modified. First of all, we posted recruitment information to further modify the initial scale through “Credamo”. A total of 30 people agreed and 12 people refused to participate. The response rate was 71%. Among them, 6 (20%) participants had a high school degree or below, 8 (27%) participants had an associate college degree, 11 (37%) participants had a bachelor’s degree, and 5 (17%) participants had a master’s degree or above. Then, the initial questionnaires were distributed to 30 people who agreed to give us suggestions on revision, including the comprehensibility, reality and accuracy of the wording about items. Voice calls and WeChat text messages were used to contact them. According to their opinions, the six-item home-based exercise scale was finally determined.

#### 3.1.2. Reliability and Validity Test

Due to the COVID-19 pandemic, a safe distance should be maintained between people. Questionnaires could not be collected face to face. This study used an online survey to collect data by “Credamo”. A total of 80 questionnaires were randomly distributed, and 63 valid questionnaires were recovered. There were 34 (54.0%) males and 29 (46.0%) females; 24 (38.1%) participants were under 25 years old, 29 (46.0%) participants were between 26–40 years old, and 10 (15.9%) participants were between 41–60 years old; 16 (25.4%) participants were students, 28 (44.4%) participants were staffs, 6 (9.5%) participants were retirees, and 13 (20.6%) participants engaged in other occupations; 23 (36.5%) participants came from cities, 15 (23.8%) participants came from counties, 18 (28.6%) participants came from towns, and 7 (11.1%) participants came from villages. To ensure the reliability and validity of the scale, exploratory factor analysis (EFA) was conducted on the sample. The results show that: Firstly, there is only one factor whose eigenvalue is greater than 1.0; secondly, the factor loading of the six items is greater than 0.6; thirdly, the explanatory power of factor to the total variance is 60%; fourthly, the reliability of factor is 0.867. The above shows that home-based exercise is a single-dimensional factor, and it has good reliability and validity (see Table 1).

### 3.2. Sample and Procedures

This study was funded by National Natural Science Foundation of China (ethical clearance number 71804119). Participants were informed and agreed to take part in this study. The sample collection process is as follows. First, sample size was determined. Online R software was used to measure the minimum sample size [61]. A power analysis for the not close model fit (α = 0.05; statistical power = 0.8; null root mean square error of approximation (RMSEA) = 0.05; and alternative RMSEA = 0.08 [62]; *df* = 129) showed the minimum sample size of 139. Second, questionnaires were collected. On “Credamo” platform, we set each participant to fill in the questionnaire only once, and a total of 514 questionnaires were collected. Third, we conducted questionnaire screening. The questionnaires filled in less than 3 min were discarded, because participants may not fill in the questionnaire when the time is too short, and questionnaires with identical answers were also invalid. Finally, 449 valid questionnaires were selected in this study, accounting for 87.35%. The sample size was over 139, which satisfies the quantity requirement, and it had an adequate level of statistical power which was over 0.99.

The questionnaires came from 27 provincial-level administrative regions including Beijing, Shanghai, Heilongjiang, Zhejiang and Xinjiang, among which Fujian, Sichuan, Guangdong and Shandong dominated. Participants were distributed in administrative regions at all levels. A total of 289 (64.4%) participants came from cities, 78 (17.4%) participants came from counties, 53 (11.8%) participants came from towns, and 29 (6.5%) participants came from villages. Regional differences ensured the universality of the questionnaire data. Overall, 194 (43.2%) participants were male, and 255 (56.8%) participants were female. Among them, 238 (53.0%) participants were unmarried, and 211 (47%) participants were married. In terms of age distribution, participants between 18–25 years old and between 26–40 years old were in the majority, accounting for 41.4% and 45.2%, respectively; 2 (0.4%) participants were under 18 years old, and 60 (13.4%) participants were between 41–60 years old. In terms of occupations, 103 (22.9%) participants were students, 284 (63.3%) participants were staffs, 31 (6.9%) participants were retirees, and 31 (8.9%) participants engaged in other occupations. In terms of the income distribution, 135 (30.1%) participants’ salary didn’t exceed CNY 3000, 169 (37.6%) participants’ salary had a salary between CNY 3001–6000, 107 (23.8%) participants had a salary between CNY 6001–10,000, and 38 (8.5%) participants’ were more than CNY 10,000. In terms of educational background, 118 (26.3%) participants had an associate college degree or below, 268 (59.7%) participants had a bachelor’s degree, and 63 (14.0%) participants had a master’s degree or above.

### 3.3. Measures

To ensure the reliability and validity of the scale, most of the international scales (except for the variable “home-based exercise”) were adopted in this study. Meanwhile, according to the needs of the study, the existing scales have been modified to a certain extent, and the reliability of the revised questionnaire was acceptable. All variables were measured on a 5-point Likert scale ranging from 1 (strongly disagree) to 5 (strongly agree).

#### 3.3.1. Health Consciousness

A five-item scale by Dutta-Bergman was applied [10]. Items included “Living life in the best possible health is very important to me”, “Eating right, exercise, and taking preventive measures will keep me healthy for life”, “My health depends on how well I take care of myself”, etc. To test people’s health consciousness during the COVID-19 pandemic, the prefix “during the COVID-19 pandemic, I felt...” was added, such as, “during the COVID-19 pandemic, I felt that living life in the best possible health is very important to me.” The Cronbach’s alpha for this scale was 0.835.

#### 3.3.2. Health Life Goal

Ingledew et al. developed life goals scale which was divided into wealth, fame and image, growth, relationships, community, and health [63], according to the Aspirations Index [41]. Health life goal is one of the dimensions, including five items, such as “The goal of my exercise is to be physically healthy”, “The goal of my exercise is to be relatively free from sickness”, etc. The Cronbach’s alpha for this scale was 0.881.

#### 3.3.3. Perceived Behavioral Control

The perceived behavioral control scale was compiled by Rhodes and Courneya [64] according to the suggestion of Ajzen [65]. This scale had three items, such as, “How confident are you over the next two weeks that you could exercise regularly if you wanted to do so”, “How much personal control do you feel you have over discipline regularly in the next two weeks”. The Cronbach’s alpha for this scale was 0.806.

#### 3.3.4. Home-Based Exercise

Home-based exercise used a six-item scale developed by us (see 3.1). Items included “During the prevention of COVID-19, I often have been doing proper exercise at home”, “During the prevention of COVID-19, I often do gymnastics at home (such as dance, yoga, aerobics, etc.)”, etc. Its Cronbach’s alpha was 0.861.

#### 3.3.5. Control Variables

The paper is a study of home-based exercise based on the COVID-19 pandemic. Therefore, people’s cognition of the COVID-19 pandemic and their attitude towards home-based exercise might affect home-based exercise. In addition, people of different ages and constitutions might have different home-based exercise. Therefore, four control variables were used, such as, “Age”, “Pandemic knowledge (i.e., How much do you know about COVID-19)”, “Perception of the importance of exercise in prevention of COVID-19 (called importance of exercise)” and “Personal health status (i.e., How do you feel about your health status)”.

### 3.4. Data Analysis

This paper used AMOS 24.0 and SPSS 22.0 to conduct the data analysis. First, confirmatory factor analysis (CFA) were adopted to test the discriminant validity between variables through AMOS 24.0. Second, this paper conducted correlation analysis and differential analysis. Third, this paper used hierarchical regression analysis proposed by Baron and Kenny [66], Structural Equation Modelling, and Hayes’ SPSS PROCESS macro [67] to test hypotheses.

## 4. Results

### 4.1. Confirmatory Factor Analysis

The model fitting standard was as follows: $\chi^{2}/df<3.0$***;*** goodness-of-fit index (GFI) > 0.80 [68]; adjusted goodness-of-fit index (AGFI) > 0.80 [69]; Tucker–Lewis index (TLI) > 0.90; comparative fit index (CFI) > 0.90; normed fit index (NFI) > 0.90 and root mean square error of approximation (RMSEA) < 0.08 [70]. According to the above criteria, the paper compared the four-, three-, two- and single-factor model, and the results showed that the four-factor model ($\chi^{2}/df=2.985$; GFI = 0.912; AGFI = 0.883; TLI = 0.928; CFI = 0.939; NFI = 0.912; RMSEA = 0.067) is the most consistent with the standard, that is, the model fitting degree is the best (see Table 2).

### 4.2. Descriptive Statistics and Differential Analysis

Descriptive statistics of the variables and correlations were shown in Table 3. There were significant positive correlations between health consciousness and health life goal (r = 0.580, *p* < 0.001), perceived behavioral control (r = 0.468, *p* < 0.001), home-based exercise (r = 0.514, *p* < 0.001). There was a significant positive correlation between health life goal and perceived behavioral control (r = 0.429, *p* < 0.001), home-based exercise (r = 0.351, *p* < 0.001). Perceived behavioral control was also positively correlated with home-based exercise (r = 0.650, *p* < 0.001). In the control variable, the age (r = 0.129, *p* < 0.001), pandemic knowledge (r = 0.321, *p* < 0.001), perception of the importance of exercise in prevention of COVID-19 (r = 0.385, *p* < 0.001) and personal health status (r = 0.487, *p* < 0.001) were significantly positively related to home-based exercise. Besides, the average variance extracted (AVE) of each variable was no less than 0.5, and the composite reliability (CR) exceeded 0.8 [71], indicating that the convergent validity and construction reliability of variables were good.

Emerging adulthood is a stage of the lifespan between adolescence and fully fledged adulthood encompassing the mid to late 20s [72]. Therefore, 25 years old was selected as the age segmentation point for *t*-test, that is, age divided into the young group (≤25 years old), and older group (>25 years old). Differential analysis showed that there were significant differences in home-based exercise with respect to gender (M _male_ = 3.92 vs. M _female_ = 3.67, t = 3.12, *p* < 0.01), age (M _young_ = 3.57 vs. M _older_ = 3.92, t = −4.26, *p* < 0.001), and marital status (M _unmarried_ = 3.66 vs. M _married_ = 3.91, t = −3.14, *p* < 0.01). Male, married and more than 25-year-old participants were more inclined to do home-based exercise during the pandemic (see Table 4).

### 4.3. Hypothesis Testing

**Figure 2:**
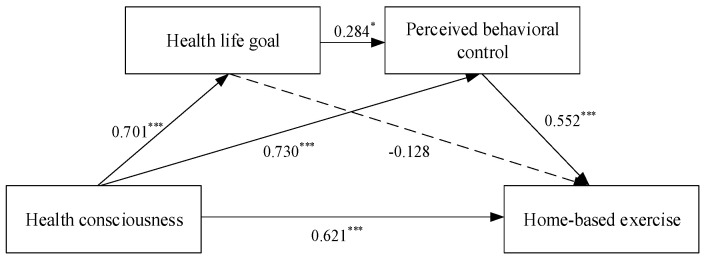
Structural model results. Notes: *n* = 449; * *p* < 0.05, *** *p* < 0.001

According to the suggestions of Baron and Kenny, this study used the method of hierarchical regression analysis to explore the influence of each variable on the dependent variable. To examine hypothesis 1, this study first set health consciousness as an independent variable, then added control variables, finally, put the independent variables (health consciousness) into the regression equation. As the results shown in Table 5 demonstrate, health consciousness had a significant positive effect on home-based exercise (M7, β = 0.311, *p* < 0.001). Hypothesis 1 was verified. Further, Structural Equation Modelling was used to test hypothesis. The full model path coefficients (γ) were shown in Figure 2. The results showed that health consciousness had a significant positive effect on home-based exercise (γ = 0.621) and it also confirmed hypothesis 1.

To verify the mediating effect, this study adopted the Hayes’ SPSS PROCESS macro (Model 6). Through it, each mediating effect could be verified. Model 6 allowed up to four mediators operating in serial. In this study, a dual mediation model was used, with a 95% confidence interval and the sample size of 5000. Table 5 showed that the indirect effect of Ind 1 was not significant, and the confidence interval of 95% bootstrap was (−0.143)–0.064 (b = −0.048, SE = 0.053, 95% CI = [−0.143, 0.064]) including 0. Therefore, hypothesis 2 was not valid, which indicated that health life goal had no mediating effect between health consciousness and home-based exercise. Ind 2 had a significant indirect effect, with 95% bootstrap confidence interval of 0.178–0.454 (b = 0.207, SE = 0.070, 95% CI = [0.178, 0.454]), not including 0; therefore, hypothesis 3 was verified. The indirect effect of Ind 3 was significant, with the confidence interval of 95% bootstrap ranging from 0.050 to 0.209 (b = 0.128, SE = 0.041, 95% CI = [0.050, 0.209]), indicating that health consciousness and home-based exercise had a chain mediation between health life goal and perceived behavioral control. Hypothesis 4 was verified (see Table 6).

## 5. Discussion

### 5.1. Theoretical Contributions

Firstly, this paper takes a survey to measure home-based exercise during the COVID-19 pandemic. In previous studies, home-based exercise was mostly used as a health intervention by scholars. For example, Kruger et al. improved the peak aerobic capacity of patients with cirrhosis by using home-based endurance exercise training [57]. Lauzé et al. discussed the feasibility and effect of the application of home-based exercise programs in the elderly [58]. This paper takes a feasible home-based exercise survey for the general population, which bases on the exercise items people can do at home during the COVID-19 pandemic. It expands research on home-based exercise, and provides reference for the survey of home-based exercise in the future.

Secondly, this paper finds that there are significant differences in home-based exercise among different categories of people. Male, married and more than 25-year-old participants are more likely to do home-based exercise during the pandemic. Some studies have also demonstrated that men participated more in regular exercise than women [73,74]. The possible explanation is that men’s athletic ability is usually better than women’s, and men are more likely to enjoy exercise in which they can gain mastery and strength [74,75]. The results showed that married people tend to exercise at home. People with a spouse may be easier to find a partner to exercise together [74]. In addition, married people need to do more physical activities at home, such as housework. People over 25 years old are more inclined to do home-based exercise, possibly because they pay more attention to a healthy lifestyle. This paper elucidated the demographic differences of home-based exercise, and laid a foundation for future studies.

Thirdly, this paper shows that health consciousness is an important driver of home-based exercise. In previous studies, many scholars have explored the role of health consciousness in health behaviors, such as buying healthy food and seeking medical services [14,15,16,18,20,21]. However, few studies have focused health behaviors on home-based exercise, especially the relationship between health consciousness and home-based exercise during the COVID-19 pandemic. The results of the study showed that during the pandemic of COVID-19, people’s health consciousness had a significant positive effect on home-based exercise. That is, in the face of a dangerous virus, the more health-conscious people are, the more likely they are to exercise at home to protect their health. This study well verified the significant influence of health consciousness on home-based exercise, and confirmed the positive effect of health consciousness on health behaviors [76,77].

Fourthly, this paper confirms the mediating effect between health consciousness and home-based exercise. Scholars usually regard perceived behavioral control as the medium between variables. Bailis et al. concluded that part of the influence of social and economic status on self-rated health was generated by perceived behavioral control through an empirical study [51]. Similar to the conclusion of Bailis et al. [51], this study found that some effects of health consciousness on home-based exercise were via perceived behavioral control. This finding extends previous studies. However, health life goal has no mediating effect between health consciousness and home-based exercise; mainly there is no significant relationship between health life goal and home-based exercise, which is inconsistent with the hypothesis. Williams et al. and Piko thought life goals had a positive impact on health behaviors [43,44], while the results of this study are not consistent with their conclusions. The reasons might be that the life goal in Williams et al. and Piko’s studies was mainly internal life goal, which included growth, relatedness, helpfulness, and health [43,44]. However, this paper only selects the dimension of health to explore. In addition, the behavior studied by Williams et al. is health-compromising behaviors (smoking) [43], while Piko studies smoking, drinking, drug use (as health-compromising behaviors), physical activity, and diet control (as health-enhancing behaviors) [44]. This paper focuses on home-based exercise during the pandemic of COVID-19. In a particular case, such as the COVID-19, people’s goal of healthy life may not be translated into home-based behavior.

Finally, this study has found an internal mechanism between health consciousness and home-based exercise. Health consciousness can affect home-based exercise through perceived behavioral control. More importantly, there is chain mediation between health consciousness and home-based exercise. People with high health consciousness will set health life goal, and then strengthen perceived behavioral control, which will eventually have a significant effect on home-based exercise. Although life goals have been demonstrated to be related to individual health behaviors [43,44], few previous studies have identified which part of life goals are most relevant to health behaviors. Often, scholars explore life goals as a whole. This study regards health life goal as an individual and explores its important role between health consciousness and home-based exercise. Overall, this study refines and deepens the related studies on health consciousness.

### 5.2. Practical Implications

The COVID-19 pandemic has made people pay more attention to personal health problems, and people’s health consciousness has been greatly improved. During the quarantine, people’s sedentary time increases with social distance [7], which may lead to health problems [4,5,6,7]. Home-based exercise, as an important way to improve physical quality and get rid of the restrictions of the environment [34], has become a national health activity during the pandemic.

This paper provides a healthy lifestyle. Home-based exercise is particularly appropriate during the pandemic. On the one hand, people should pay attention to their personal health problems and improve their health consciousness. The results show that health consciousness has a very positive impact on home-based exercise. Improving personal health consciousness is conducive to exercise at home and promote health. On the other hand, people should translate health consciousness into behaviors that actually improve health. According to this study, health consciousness can promote the formation of health life goal, further enhance perceived behavioral control, and ultimately promote people to implement home-based exercise during the COVID-19 pandemic. Based on this, people should attach importance to the setting of health life goal. Once the goals are formed, they will have a sense of direction and guide people to accomplish them. Besides, people cannot ignore the belief that individuals control behaviors. Home is a comfort zone and it is easy to relax for people in this environment. Previous research shows that time at home is mostly inactive for adolescents [78], and they tend to engage in sedentary behaviors at home, such as sleeping, watching TV, and playing online games [5]. Therefore, self-control is very important in the home environment, and it can drive people’s behaviors well. The stronger perceived behavioral control, the more actively people engage in home-based exercise. Home-based exercise is not restricted by environmental factors. In the future, people can also adopt this way to achieve health life goal. In a word, this study has a strong practical significance; people should have direction and confidence to fight against the health crisis.

## 6. Limits and Suggestions for Future Studies

This study reveals the intrinsic link between health consciousness and home-based exercise during the COVID-19 pandemic, but it still has some limitations. First, due to the pandemic, this study can only collect questionnaires on an online platform. Although the study controls the quality of the questionnaires by screening questionnaires, the real situation of the respondents filling in the questionnaire could not be understood. In addition, respondents are restricted to those who are able to fill out an online questionnaire. Future studies can collect questionnaires by combining online and offline methods. Second, the age of participants is mainly concentrated between 18 and 40 years old. People in this age group usually feel in good health. Therefore, it happened that age was positively correlated with personal health status. Future studies should expand the distribution of age. Third, different populations have different home-based exercise habits. This paper found the significant differences of people participating in home-based exercise in terms of gender, age, and marital status. However, this paper did not explore the impact of demographic factors on the internal mechanism of home-based exercise. In the future, we can explore the influence of demographic factors such gender, age, socioeconomic status on the internal mechanism of home-based exercise. Fourth, this study explored the mediating effect of health life goal and perceived behavioral control on health consciousness and home-based exercise. However, the influence paths of health consciousness on home-based exercise are varied, among which there are other mediating effects. Future study can further explore the mediating effect of factors such as self-efficacy and attitude between health consciousness and home-based exercise. Fifth, this paper just focuses on the influence of health consciousness, health life goal and perceived behavioral control for home-based exercise. There may be other factors that influence home-based exercise, such as perceived usefulness. People think that the more useful home-based exercise is, the more likely they are to exercise at home. The following studies can deeply explore the other influencing factors (e.g., perceived usefulness, self-efficacy, etc.) of home-based exercise. Furthermore, the cross-sectional data used in this study cannot reflect the dynamic process of the internal mechanism of home-based exercise with the evolution of pandemic. Further studies using longitudinal data may yield new findings.

## 7. Conclusions

This study mainly discussed the influencing mechanism of health consciousness on home-based exercise during the pandemic of COVID-19. Health consciousness can not only directly affect home-based exercise, but also indirectly affect home-based exercise through health life goal and perceived behavioral control. Perceived behavioral control plays a mediating role between health consciousness and home-based exercise. The findings of this paper show the importance of establishing health life goal in daily life, which points out the direction for us to adopt health behaviors. In addition, people have to improve the individual control to ensure that the behaviors are carried out. Life lies in healthy exercise. Regardless of external factors, people should create favorable conditions to keep doing exercise.

## Figures and Tables

**Table 1:** Results of exploratory factor analysis result.

Items	Factor Loading	Eigenvalue
During the prevention of COVID-19, I often have been doing proper exercises at home.	0.863	3.666
During the prevention of COVID-19, I often do gymnastics at home (such as dance, yoga, aerobics, etc.).	0.883	0.968
During the prevention of COVID-19, I often walk around or jog around the house.	0.819	0.445
During the prevention of COVID-19, I often do stretching exercises at home (such as leg press, leg lift, joint movement, etc.).	0.808	0.397
During the prevention of COVID-19, I often do housework at home (such as cooking and cleaning).	0.580	0.273
During the prevention of COVID-19, I often do other exercises at home (such as tai chi, bodybuilding, strength training, etc.).	0.694	0.252
Explanatory power of the total variance (%).	61.102	
Reliability.	0.867	

Note: *n* = 63.

**Table 2:** Results of confirmatory factor analysis.

Model	$\chi^{2}$	*df*	$\chi^{2}/df$	GFI	AGFI	TLI	CFI	NFI	RMSEA
Four factor	385.079	129	2.985	0.912	0.883	0.928	0.939	0.912	0.067
Three factor	592.126	132	4.486	0.871	0.832	0.873	0.891	0.864	0.088
Two factor	940.271	134	7.017	0.778	0.717	0.781	0.808	0.784	0.116
Single factor	1645.355	135	12.188	0.614	0.511	0.593	0.641	0.622	0.158

Notes: Four-factor model considers all four variables as independent factors; Three-factor model: combining home-based exercise with perceived behavioral control; Two-factor model: combining home-based exercise with perceived behavioral control, combining health consciousness with health life goal; Single-factor model: treats all variables as one dimension.

**Table 3:** Descriptive statistics and correlation analysis.

	Age	Pandemic Knowledge	Importance of Exercise	Personal Health Status	Health Consciousness	Health Life Goal	Perceived Behavioral Control	Home-Based Exercise
Age	1.000							
Pandemic knowledge	−0.008	1.000						
Importance of exercise	0.001	0.177 ***	1.000					
Personal health status	0.100 *	0.327 ***	0.262 ***	1.000				
Health consciousness	0.069	0.226 ***	0.421 ***	0.632 ***	1.000			
Health life goal	0.057	0.260 ***	0.323 ***	0.320 ***	0.580 ***	1.000		
Perceived behavioral control	0.074	0.308 ***	0.316 ***	0.500 ***	0.468 ***	0.429 ***	1.000	
Home-based exercise	0.129 **	0.321 ***	0.385 ***	0.487 ***	0.514 ***	0.351 ***	0.650 ***	1.000
Mean	2.710	4.070	4.480	4.140	4.443	4.345	3.852	3.778
SD	0.694	0.676	0.658	0.793	0.490	0.543	0.782	0.853
AVE					0.507	0.649	0.597	0.525
CR					0.835	0.881	0.806	0.867

Notes: Age: 1 = under 18 years old, 2 = 18–25 years old, 3 = 26–40 years old, 4 = 41–60 years old, 5 = 61 years old and above; Pandemic knowledge: 1= very little, 2 = little, 3 = uncertainty, 4 = more, 5 = very much; Importance of exercise: 1 = very unimportant, 2 = not very important, 3 = uncertainty, 4 = more important, 5 = very important; Personal health status: 1 = very poor, 2 = poor, 3 = in general, 4 = better, 5 = very good; *n* = 449; * *p* < 0.05, ** *p* < 0.01, *** *p* < 0.001; CR = Composite Reliabilities; AVE = Average Variance Extracted.

**Table 4:** Differential analysis (*t*-test), according to gender, age and marital status.

*n* = 449	Male	Female	*T*-Test(*p*)	≤25 years old	>25 years old	*T*-Test (*p*)	Unmarried	Married	*T*-Test(*p*)
	*n* = 194(43.2%)	*n* = 255(56.8%)	-	*n* = 186(41.4%)	*n* = 263(58.6%)	-	*n* = 238(53.0%)	*n* = 211(47.0%)	-
	Mean(SD)	Mean(SD)	-	Mean(SD)	Mean(SD)	-	Mean(SD)	Mean(SD)	-
	3.92(0.78)	3.67(0.89)		3.57(0.90)	3.92 (0.79)		3.66(0.88)	3.91(0.80)	
Home-based exercise	-	-	3.21(0.001 **)	-	-	−4.26(0.000 ***)	-	-	−3.14(0.002 **)

Notes: ** *p* < 0.01, *** *p* < 0.001.

**Table 5:** Test results of hypothesis.

	Health Life Goal		Perceived Behavioral Control			Home-Based Exercise		
	M1	M2	M3	M4	M5	M6	M7	M8
**Control variables**								
Age	0.037	0.015	0.035	0.022	0.02	0.094 *	0.080 *	0.071 *
Pandemic knowledge	0.150 **	0.107 **	0.144 **	0.120 **	0.103 *	0.159 ***	0.132 **	0.081 *
Importance of exercise	0.243 ***	0.074	0.186 ***	0.089 *	0.078	0.264 ***	0.157 ***	0.120 **
Personal health status	0.203 ***	0.085 *	0.401 ***	0.334 ***	0.320 ***	0.356 ***	0.282 ***	0.141 ***
**Independent variables**								0.001
Health consciousness		0.492 ***		0.281 ***	0.205 ***		0.311 ***	0.192 ***
**Mediating variables**								
Health life goal					0.155 **			
Perceived behavioral control								0.421 ***
**R^2^**	0.185	0.365	0.306	0.365	0.38	0.338	0.41	0.523
**F**	25.140 ***	51.023 ***	48.999 ***	50.956 ***	45.225 ***	56.714 ***	61.617 ***	80.712 ***
**ΔR^2^**	0.185	0.181	0.306	0.059	0.015	0.338	0.072	0.113
**ΔF**	25.140 ***	126.201 ***	48.999 ***	41.086 ***	10.884 **	56.714 ***	54.099 ***	104.326 ***

Notes: *n* = 449; * *p* < 0.05, ** *p* < 0.01, *** *p* < 0.001.

**Table 6:** Mediation Model: Indirect effect between health consciousness and home-based exercise through health life goal and perceived behavioral control.

	b	SE	Bootstrap 95% CI
Total Effect	0.895	0.071	[0.756, 1.049]
Direct Effect	0.509	0.076	[0.358, 0.659]
Indirect Effect	0.386	0.073	[0.245, 0.536]
Ind 1: HC→HLG→HBE	−0.048	0.053	[−0.143, 0.064]
Ind 2: HC→PBC→HBE	0.307	0.070	[0.178, 0.454]
Ind 3: HC→HLG→PBC→HBE	0.128	0.041	[0.050, 0.209]

Notes: Choosing Model 6 in the PROCESS macro; b is the unstandardized regression coefficients; SE is the standard errors; CI is the confidence intervals; HC = Health consciousness; HLG = Health life goal; PBC = Perceived behavioral control; HBE = Home-based exercise.
